# Evaluation of early complications, outcome, and mortality in Coronavirus Disease 2019 (COVID-19) infection in patients who underwent orthopedic surgery

**DOI:** 10.1186/s12891-022-05010-8

**Published:** 2022-01-18

**Authors:** Mehdi Mohammadpour, Hamidreza Yazdi, Abolfazl Bagherifard, Mahmoud Jabalameli, Mehdi Moghtadaei, Ali Torkaman, Hooman Yahyazadeh, Mohammad Taher Ghaderi, Mohammad Mahdi Fanaeian, Moein Khaleghi Langeroudi, Peyman Hashemi, Saeed Razi, Amer Karimpour, Sam Bemani Lirgeshasi, Milad Bahari

**Affiliations:** 1 Bone and Joint Reconstruction Research Center, Department of Orthopedics, School of Medicine, Iran University of Medical Sciences, Tehran, Iran; 2 Division of Gastroenterology and Liver Diseases, Imam Khomeini Hospital Complex, Tehran University of Medical Sciences, Tehran, Iran

**Keywords:** COVID-19, Orthopedic procedures, Threshold, Mortality

## Abstract

**Background:**

A higher mortality and morbidity rate has been reported in COVID-19 patients undergoing surgery. To reduce the morbidity and mortality rate in COVID-19 patients undergoing orthopedic procedures, we aimed to increase the threshold for surgical planning.

**Methods:**

In a prospective cohort study, COVID-19 patients who underwent elective or emergent orthopedic surgery in three orthopedic surgery centers from February 2020 to September 2020 were included. In this period, 6751 patients were scheduled for orthopedic surgery. To increase surgical threshold planning, all patients with grade 5 of the American Society of Anesthesiologists (ASA) classification and patients with COVID-19 related moderate to severe pulmonary involvement were identified as high-risk patients and were excluded.

**Results:**

35 deaths occurred during the study. The frequency of deaths was significantly higher in patients with COVID-19, 6 (9.4%) than patients without COVID-19, 29 (0. 43%). The average hospitalization stay was 12.8 ± 12.1 days. The odds ratio (OR) for death was significantly higher in patients with COVID-19 than patients without COVID-19. [OR: 8.13, Confidence interval 95% (CI95%) (5.02–11.25), P: 0.001]. Four (6.3%) COVID-19-associated complications were recorded in this series that all were respiratory failure requiring unexpected postoperative ventilation. Twenty surgical complications (31.3%) were recorded. The odds ratio for ICU admission was significantly higher in patients with COVID-19 than patients without COVID-19. [OR: 5.46, CI 95% (2.68–8.68), P: 0.001].

**Conclusions:**

An increased threshold for orthopedic surgery is suggested for COVID-19 patients with a mortality rate of 9.3%, which is less than the mortality rate in other studies.

Level of evidence III.

**Supplementary Information:**

The online version contains supplementary material available at 10.1186/s12891-022-05010-8.

## Background

The COVID-19 pandemic, caused by severe acute respiratory syndrome virus-2 (SARS-CoV-2), has turned into the biggest challenge of the twenty-first century [1]. This challenge is even bigger for vulnerable populations at a higher risk of COVID-19 morbidities and mortality [2]. Old age, obesity, comorbidities such as diabetes mellitus, chronic obstructive pulmonary disease (COPD), chronic kidney disease, severe cardiovascular diseases (e.g., heart failure and coronary artery disease), cancer, the immunocompromised state in solid organ transplant, and smoking are acknowledged as the established risk factors for severe COVID-19 infection and subsequent increased mortality [3–6].

Patients infected with COVID-19 who undergo surgery are prone to subsequent pulmonary complications because of the release of the pro-inflammatory cytokines, immunosuppressive responses to surgery, and mechanical ventilation [7]. A higher rate of mortality and pulmonary complications in COVID-19 patients undergoing surgery has been reported in several studies, which are 21.1 to 32.4% [8–11].

Considering the higher risk of mortality in COVID-19 patients undergoing surgery, the risks and benefits of surgery in these patients should be thoroughly assessed. For this purpose, surgery-specific risk evaluation of COVID-19 morbidity and mortality is of significant value [8].

To reduce the mortality and morbidity rate in patients undergoing orthopedic procedures, we aimed to increase the threshold for orthopedic surgical planning. We hypothesized that this modification might reduce the rate of mortality.

## Methods

The ethics committee approved this multi-center prospective study of our institute. Participants signed a written informed consent to use their medical data for publication. Between February 20 and September 20, 2020, 6751 patients who underwent orthopedic surgery were included in the study. Patients were then divided into two groups based on the presence or absence of the COVID-19 infection virus. Group 1 was the patients who underwent surgery and were infected with the COVID-19 infection virus. Group 2 patients underwent orthopedic surgery and were not infected with the COVID-19 infection virus. Data were collected using a checklist and from patients’ medical records. Patients infected with COVID-19 who underwent elective or emergency surgery in three hospitals were prospectively evaluated for eligibility criteria. The inclusion criteria were; the age of > 18 years, non-pregnant women, a definite diagnosis of COVID-19 by Real-Time RNA specific Reverse Transcriptase Polymerase Chain Reaction (RT-PCR) or computed tomography (CT) scan imaging (pneumonia), and oxygen saturation of 94% or less [12]. diagnosis of COVID-19 by a senior physician specialized in infectious diseases and a minimum postoperative follow-up of 30 days. The diagnosis was based on clinical presentations suggestive of COVID-19 infection [13]. The findings of CT were described as bilateral lung opacities and lobular and sub-segmental areas of consolidation [14].

**Fig. 1:**
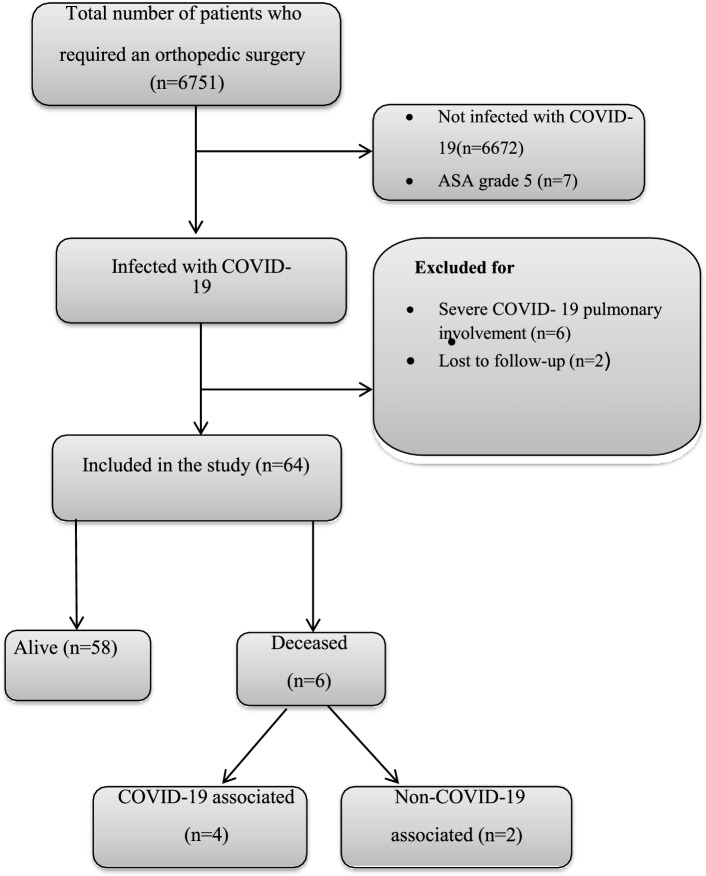
Flowchart of patient inclusion and exclusion in the study

We opted for an increased surgical threshold during the COVID-19 pandemic so that surgical procedure for high-risk patients was postponed as much as possible. In this respect, patients with grade 5 of the ASA classification were excluded in both groups from the beginning of the study (*n* = 7), patients with moderate to severe COVID-19 pulmonary involvement (lung involvement equal or more than 50%) [14] (*n* = 6), were identified and excluded (Appendix 1). two patients were also excluded due to loss of follow-up (*n* = 2) (Fig. 1).

Finally, 64 patients with COVID-19 and 6682 without COVID-19 who underwent orthopedic surgery during the study period were enrolled. For emergent cases with a clinical diagnosis of COVID-19, a chest x-ray (CXR), chest computed tomography (CT) scan, and laboratory tests including complete blood count (CBC), Erythrocyte Sedimentation Rate (ESR), and C-reactive protein (CRP) were done.

RT-PCR (Reverse Transcription Polymerase Chain Reaction) test was performed on 44 patients. PCR test was positive for 20 patients and harmful for 24 patients. In contrast, no PCR was performed for 20 patients. We confirmed the clinical diagnosis of COVID-19 with RT-PCR in 20 cases. As mentioned in our study, 20 patients were diagnosed when our center was not equipped with this laboratory test for PCR diagnostic test of this disease. PCR test was only available about 4 months after the start of the COVID-19 pandemic that was based on the detection of viral Ribonucleic acid (RNA) in samples obtained from nasal swabs or bronchoalveolar lavage.. At the last follow-up, antibody tests were performed on all cases. The result of the antibody test was positive for all patients.

Patient demographics, clinical, laboratory, surgical, and hospitalization data were recorded. The primary outcome of interest was mortality within 30 days after the surgery. The secondary outcome of interest was complications within 30 days past the surgery. Two groups were compared in terms of outcomes.

### Statistical analysis

The mean and standard deviation (SD) were conducted for the descriptive analysis of continuous variables and qualitative data; the descriptive analysis was performed using frequencies. We used Kolmogorov–Smirnov and Shapiro-Wilk normality tests, P-P plot, and histogram to test the normality of our study population. For the continuous variables, a t-test was employed to analyze the difference; however, the Mann-Whitney U test was used in non-parametric conditions. A chi-squared test (χ2 test) was conducted for the classified variables. A two-tailed *P* value of < 0.05 was considered statistically significant. Multivariate logistic regression analysis was used to control confounding variables. Variables with *P* < 0.10 in the univariate analysis were entered into a multivariate logistic regression analysis using the backward selection method. The odds ratio adjusted was used to report the results. The statistical analyses were performed using the SPSS 20.0 (SPSS Inc) software.

## Results

6751 patients were enrolled (64 patients with COVID-19, 6682 patients without COVID-19, and 7 patients with grade 5 of the ASA). Overall, the mean age of patients was 50.25 ± 18.91 years.83.4% were male, and 16.6% were female. 48% of patients had at least one underlying disease. 24% of patients had hypertension, and 14% had type 2 diabetes. Overall, 22% of patients had a history of smoking. Overall, the mean follow-up period was 114.6 ± 63.3 days. The mean follow-up period in patients with and without COVID-19 was 115.3 ± 63.6 and 114.75 ± 66.5 days, respectively. No significant differences were observed for the demographic characteristics of the patients in the two groups. (*p* > 0.05) Table 1.

**Table 1:** Comparison Baseline characteristics of study population

Variable	Patients with COVID-19 (***n*** = 64)	Patients without COVID-19 (***n*** = 6682)	***P*** value
**Age (year)**	51.3 ± 19.5	50.19 ± 18.88	0.87
**Gender**			0.91
• **Male**	54 (84.4)	5553 (83.1)	
• **Female**	10 (15.6)	1129 (16.9)	
**Body mass index (kg/m**^**2**^**)**	25.5 ± 5.3	25.2 ± 5.01	0.85
**Education**			0.62
• **Less than high school**	25 (39.1)	2573 (38.5)	
• **High school diploma**	30 (46.8)	3247 (48.6)	
• **Academic education**	9 (14.1)	862 (12.9)	
**Current smoker**			0.12
• **Yes**	15 (23.4)	1450 (21.7)	
• **No**	49 (76.6)	5232 (78.3)	
**Drug abuser**			0.96
• **Yes**	14 (21.9)	1457 (21.8)	
• **No**	50 (78.1)	5225 (78.2)	
**Alcoholic**			0.16
• **Yes**	3 (4.7)	341 (5.1)	
• **No**	61 (95.3)	6341 (94.9)	
Comorbidity			0.11
• None	33 (51.5)	3481 (52.1)	
• Respiratory	3 (4.6)	274 (4.1)	
• CVD	10 (15.6)	1076 (16.1)	
• CNS	1 (1.6)	94 (1.4)	
• Malignancy	1 (1.6)	94 (1.4)	
• DM	2 (3.1)	231 (3.46)	
• Psychiatry	2 (3.1)	201 (3)	
• CVD & renal	1 (1.6)	98 (1.46)	
• CVD & DM	6 (9.3)	635 (9.5)	
• Respiratory + CVD + CNS	1 (1.6)	140 (2.1)	
• Respiratory + CVD + DM	1 (1.6)	90 (1.35)	
• CVD + Renal + CNS	1 (1.6)	97 (1.45)	
• CVD + CNS + DM	1 (1.6)	99 (1.49)	
• Respiratory + CNS + Malignancy	1 (1.6)	72 (1.09)	
Mean follow-up (day)	115.3 ± 63.6	114.4 ± 64.1	0.49

Data are presented as mean ± SD or number & %

*DM* Diabetes mellitus, *CVD* Cardiovascular disorders, *GI* Gastrointestinal disorders, *CNS* Central nervous system disorders

Elective patients diagnosed with COVID-19 developed COVID-19 after surgery and even after discharge and were not infected before surgery. The majority of the patients (34.3%) were asymptomatic; then, fever was the main clinical symptom of COVID-19 that was present in 30 (46.8%) patients alone or combined with other clinical symptoms (Table 2). As mentioned, several patients were asymptomatic. Because, before surgery, all patients underwent screening with a preliminary laboratory examination and lung CT scan.

**Table 2:** Characteristics of the COVID-19 in patients who underwent orthopedic procedures

Variable	Patients (*n* = 64)
**Clinical symptom**	
1. None	22 (34.3)
2. Dyspnea	7 (10.9)
3. Cough	3 (4.7)
4. Fever	13 (20.3)
5. Dyspnea & Cough	2 (3.1)
6. Dyspnea & fever	6 (9.4)
7. Fever & Cough	6 (9.4)
8. Fever & Fatigue	1 (1.6)
9. Dyspnea & fever & cough	2 (3.1)
10. Fever & Fatigue & cough	1 (1.6)
11. Dyspnea & fever & cough & Fatigue & GI symptom	1 (1.6)
**Preoperative respiratory support**	
• No	61 (95.3)
• None Invasive	2 (3.1)
• Invasive	1 (1.6)
**Chest radiograph**	
• Positive	40 (62.5)
• Negative	24 (37.5)
**Chest CT scan**	
• Positive	57 (89.1)
• Negative	7 (10.9)
**Leucopenia**	
• Yes	3 (4.7)
• No	61 (95.3)
**Lymphopenia**	
• Yes	22 (34.4)
• No	42 (65.6)
**PCR**	
• Positive	20 (31.2)
• Negative	24 (37.6)
• Not performed	20 (31.2)
**ESR**	
• Elevated	44 (68.8)
• Normal	20 (31.2)
**CRP**	
• Elevated	54 (80.8)
• Normal	10 (19.2)
**Normal INR (< 1.1)**	
• Yes	44 (68.8)
• No	20 (31.2)

*GI* Gastrointestinal, *PCR* Polymerase chain reaction, *ESR* Erythrocyte sedimentation rate, C-reactive protein, *INR* International normalized ratio

Overall, the surgery was emergent in most patients 89.1% in patients with COVID-19 and 88.3% in patients without COVID-19). This difference was not statistically significant in the two groups. (p:0.69) ASA grade 2 was the most frequent grade of anesthesia, 77.6%. The lower extremity was the most frequent site of surgery 41.8%. No statistically significant differences were observed for Priority, Involve limb, Type of surgery, ASA grade, and Type of anesthesia in the two groups. (*p* > 0.05) (Table 3).

**Table 3:** Comparison of surgical characteristics in patients with and without COVID-19 who underwent orthopedic procedures

Variable	Patients with COVID-19 (***n*** = 64)	Patients without COVID-19 (***n*** = 6682)	***P*** value
**Priority**			0.69
• **Emergency**	57 (89.1)	5900 (88.3)	
• **Elective**	7 (10.9)	782 (11.7)	
**Involve limb**			0.71
1. **Upper extremity**	13 (20.3)	1383 (20.7)	
2. **Lower extremity**	27 (42.1)	2787 (41.7)	
3. **Spine**	3 (4.7)	321 (4.8)	
4. **Hip**	17 (26.5)	1784 (26.7)	
5. **Upper & lower extremity**	1 (1.6)	113 (1.7)	
6. **Hip & lower extremity**	1 (1.6)	98 (1.5)	
7. **Spine & lower extremity**	1 (1.6)	95 (1.4)	
8. **Upper & lower extremity & hip**	1 (1.6)	101 (1.5)	
**Type of surgery**			0.81
1. **CR & Immobilization**	3 (4.7)	325 (4.9)	
2. **CR & PCP**	3 (4.7)	318 (4.6)	
3. **Plating**	26 (40.6)	2740 (41)	
4. **IMN**	7 (10.9)	690 (10.4)	
5. **Arthroplasty**	3 (4.7)	318 (4.8)	
6. **Spine surgery**	3 (4.7)	309 (4.6)	
7. **Soft-tissue surgery**	15 (23.4)	1561 (23.4)	
8. **CR & plating**	2 (3.1)	198 (3)	
9. **Plating & IMN**	1 (1.6)	101 (1.5)	
10. **Plating & spine surgery**	1 (1.6)	122 (1.8)	
**ASA grade**			0.61
• **1**	3 (4.7)	328 (4.9)	
• **2**	50 (78.1)	5172 (77.4)	
• **3**	8 (12.5)	861 (12.9)	
• **4**	3 (4.7)	321 (4.8)	
**Type of anesthesia**			0.48
• **Regional**	35 (54.7)	3635 (54.4)	
• **General**	29 (45.3)	3047 (45.6)	

*CR* Close reduction, *PCP* Primary care providing, *IMN* Intramedullary nailing, *ASA* American Society of Anesthesiologists, *ml* milliliter

### Comparison of complications and mortality

Overall, the average hospitalization stay was 12.8 ± 12.1 days (range 1–60). Mean operation time was 169.7 ± 104.5 min (range 30–510). The results showed that 35 deaths occurred during the study. The frequency of deaths was significantly higher in patients with COVID-19 (9.4%) than patients without COVID-19; 29 (0.43%). The odds ratio adjusted (OR_adj_) for death was significantly higher in patients with COVID-19 than patients without COVID-19. [Odds ratio (OR_adj_): 8.13, Confidence interval 95% (CI95%) (5.02–11.25), P: 0.001]. Four (6.3%) COVID-19-associated complications were recorded in this series that all were respiratory failure requiring unexpected postoperative ventilation. Twenty surgical complications (31.3%) were recorded in this series, which included three cases of surgical site infection (4.7%) and 17 cases (26.6%) of stiffness. No case of DVT was recorded in our patients. The frequency of ICU admission was significantly higher in patients with COVID-19. The odds ratio (OR adjusted) for ICU admission was significantly higher in patients with COVID-19 than patients without COVID-19. [OR_adj_: 5.46, CI 95% (2.68–8.68), P: 0.001]. There was no significant difference between the length of hospital stay, Mean surgical duration, and intraoperative bleeding in the two groups. Table 4.

**Table 4:** Comparison of complications, Outcomes and mortality characteristics in patients with and without COVID-19 who underwent orthopedic procedures

Variable	Patients with COVID-19 (***n*** = 64)	Patients without COVID-19 (***n*** = 6682)	***P*** value
Mean Hospitalization (day)	13.5 ± 12.4	12.7 ± 12.1	0.43
ICU admission			0.026
• Yes	30 (46.9)	1423 (21.3)	
• No	34 (53.1)	5259 (78.7)	
Mean surgical duration (min)	171.7 ± 104.2	169.5 ± 104.1	0.39
Death			0.001
• Yes	6 (9.4)	29 (4.3)	
• No	58 (90.6)	6653 (95.7)	
Intraoperative bleeding			0.098
• < 100 ml	27 (42.2)	2666 (39.9)	
• ≥100 ml	37 (57.8)	4016 (60.1)	
Anesthesia type			0.86
• General	7 (10.9%)	641 (9.6%)	
• Regional	57 (89.1%)	6.041 (90.4%)	

## Discussion

In this prospective cohort study, we evaluated the mortality rate and complications in patients with and without COVID-19 who underwent orthopedic procedures with an increased threshold for surgical planning. In total, 20 postoperative complications occurred in this series. The overall mortality rate was 0.52% (35 out of 6746). The mortality rates in patients with and without COVID-19 were 9.4% and 0.43, respectively. The frequency of admission to ICU was significantly higher in patients with COVID-19. Four of these deaths (6.2%) were associated with COVID-19 and due to respiratory failure. All patients with COVID-19 associated deaths were over 55 years and had comorbid disorders such as diabetes mellitus (*n* = 2) and diabetes mellitus with cardiopulmonary (*n* = 2).

The influence of COVID-19 on postoperative mortality of patients undergoing orthopedic surgery has been investigated in a few numbers of other studies. Our study showed that the mortality rate in patients with COVID-19 after orthopedic surgery was significantly higher than patients without COVID-19, which was consistent with the results of previous studies in this field [15, 16]. Nepogodiev et al., in a cohort study at 235 hospitals in 24 countries, reported 30-day pulmonary complication and mortality rates in patients with perioperative COVID-19. Of 1128 patients who underwent surgery during the study period, 294 (26.1%) patients had COVID-19. The total 30-day rate of mortality was 23.8% (268 of 1128). The total 30-day rate of mortality was considerably higher in patients with pulmonary complications so that 81.7% of all deaths (219 of 268) occurred in this group. The overall 30-day rate of mortality and pulmonary complications was 21.1% (62 of 294) and 48.3 (142 of 294) in patients with a preoperative diagnosis of COVID-19. Orthopedic surgery was performed in 302 patients of this study. Eighty-six deaths were recorded in patients who underwent orthopedic surgery. Pulmonary complications occurred in 131 (44.3%) patients who underwent orthopedic surgery. Male sex, age of ≥70 years ASA grades 3–5, malignant diagnosis, emergency surgery, and major surgery were associated with a higher mortality rate. They suggested a higher threshold for surgery during the COVID-19 pandemic than normal practice, particularly for patients with mortality risk factors [8]. The threshold for orthopedic surgery during the COVID-19 pandemic was higher in our center so that we tried to treat patients conservatively as much as possible. All COVID-19 associated deaths of the present series were complicated with pulmonary complications, showing a higher mortality rate in patients with pulmonary involvement, as stated in the study of Nepogodiev et al. Knisely et al. [16], in a cohort study in 2021, evaluated 468 patients with COVID-19 who undergo urgent and emergent surgery. The results showed that 36(7.7%) subjects were infected with the COVID-19 virus. The mortality rate in COVID-19 positive subjects (16.7%) was significantly higher than COVID-19 negative subjects (1.4%). (aRR = 7.02; 95%CI, 4.96–9.92) which was consistent with the results of our study. The high number of deaths in this study compared to ours can be justified due to differences in patient characteristics and the difference in sample size in the study. This study also showed that the rate of admission to ICU was significantly higher in patients with COVID-19 (36.1%) compared to patients without COVID-19 (16.4%), which was consistent with the results of our study that the admission rate in ICU in patients with and without COVID-19 was estimated at 46.9 and 21.3%, respectively. Clement et al. evaluated the effect of COVID-19 on postoperative mortality of patients undergoing orthopedic and trauma surgery in nine centers. Of 1569 patients who underwent surgery during the study period, 68 (4.3%) were diagnosed with COVID-19. The overall rate of mortality was 5.4% (*n* = 85). Twenty-two deaths (32.4%) occurred in COVID-19 patients. The survival rate was significantly lower in patients with COVID-19 infection (67.6% vs. 95.8%). Older age, female sex, hip fracture, and increasing ASA grade increased mortality risk [10]. The mortality rate of COVID-19 patients in the present series was 9.3%, significantly lower than the study of Clement et al. This lower mortality rate could be attributed to the patients’ selection and increased threshold for surgery in the present study. Moreover, the other reason for the decrease in mortality in patients with COVID-19 may have been due to the increasing experience of physicians during this period.

While most patients in our study were ASA grade 2 (50 of 64), the majority of COVID-19 patients in the study of Clement et al. were grade 3 or 4 (56 of 68). In addition, our study’s duration was longer than the Clement study (7 months vs. 1.6 months), and more patients were evaluated (6746 vs. 1659).

Sobti et al. compared the mortality rate in patients with femoral neck fracture with or without COVID-19. From a total of 94 patients who were operated on for femoral neck fracture, nine deaths were recorded that three of them were COVID-19 positive, five were COVID-19 negative, and the other one was not tested. According to their conclusion, the mortality rate in patients undergoing surgery for femoral neck fracture was not significantly different compared to the cohort performed before COVID-19 lockdown [17]. Considering the high mortality rate of a femoral neck fracture, particularly in the geriatric population [18].

Several other studies have also studied the mortality and morbidity rate in COVID-19 patients undergoing different surgical procedures. Wang et al., in a meta-analysis study of 269 patients from 47 studies, evaluated the factors affecting the mortality of COVID-19 patients undergoing surgery. The mean age of the patients was 50.9 years. The overall rate of mortality was 6%. All deceased patients had complications associated with either surgery or COVID-19. Patients with severe respiratory complications at presentation had higher postoperative mortality [19]. These results further highlight the importance of a higher threshold for surgical procedures during the COVID-19 pandemic.

Abate et al. assessed the postoperative mortality rate in COVID-19 undergoing surgery in a meta-analysis of 33 studies with 2947 participants. According to this study, the postoperative mortality rate among COVID-19 patients was 20%. The postoperative ICU admission rate was 15% [11]. In the present study, the postoperative mortality and ICU admission rates were 9.3 and 46.9%.

Besides the increased mortality and morbidity rate in COVID-19 patients undergoing orthopedic surgeries, SARS-CoV-2 infection has other adverse impacts on the management of patients, such as in the diagnosis of postoperative infection. Since COVID-19 disease could be associated with increased ESR and CRP levels [20, 21], identification of surgical site infection in patients with COVID-19 could be challenging. In addition, the hospitalization stay of diabetic patients is a matter of concern. Based on our study, diabetic patients are one of the most vulnerable groups to COVID-19. A more extended hospitalization stay could increase the possibility of COVID-19 infection. At the same time, the patients require intensive care in the hospital, and a shorter hospital stay increases the probability of diabetic foot sepsis. Our study evaluated a large sample size of orthopedic surgeries, and multivariate logistic regression analysis was used to control confounding variables. The prospective study design was another strength of our study compared to other studies. COVID-19 mortality rates have also been reported based on underlying diseases and socioeconomic [6, 22–24]. According to our research so far, the study in Iran has not evaluated the outcomes of COVID-19 in orthopedic surgeries. Our study was conducted in a multi-center for the first time in Iran.

The present study was not without limitations. The main end of this study was the small number of COVID-19 patients, which did not allow statistical evaluation of the risk factors associated with the morbidity and mortality of COVID-19 patients undergoing orthopedic surgeries. Due to the unknown nature of this disease in the early pandemic, based on the literature and opinions of orthopedic specialists, only the factors that could worsen patients in orthopedic surgery were recorded and evaluated. Therefore, a few items related to orthopedic surgery were assessed. Therefore, future studies with larger patients’ numbers and broader factors associated with orthopedic surgery may be required for a detailed evaluation of the risk factors of morbidity and mortality in these patients. PCR test was not available from the beginning of this study, and we made the diagnosis based on laboratory findings and lung CT scan. The strength of this study was the multi-center study design and the high sample size of this study.

## Conclusion

According to our observation, Covid-19 increased the chances of mortality and ICU admission, even in patients with a higher threshold for surgery who underwent orthopedic surgery than those without Covid-19.

## Supplementary Information

**Additional file 1.**

## Data Availability

The datasets used and/or analyzed during the current study are available from the corresponding author on reasonable request.

## Abbreviations

COVID-19: Coronavirus Disease 2019
ASA: American Society of Anesthesiologists
COPD: Chronic Obstructive Pulmonary Disease
CXR: Chest X-ray
CT: Computed Tomography scan
CBC: Complete Blood Count
ESR: Erythrocyte Sedimentation Rate
CRP: C-reactive protein
RNA: Ribonucleic acid
OR: Odds Ratio
