# Supplementary material for: Evaluation of early complications, outcome, and mortality in Coronavirus Disease 2019 (COVID-19) infection in patients who underwent orthopedic surgery
Source: BMC Musculoskelet Disord. 2022 Jan 18;23:64. doi: 10.1186/s12891-022-05010-8 (PMC8764495; doi:10.1186/s12891-022-05010-8)
Supplement: Supplementary file 1 — Additional file 1. [file 12891_2022_5010_MOESM1_ESM.docx]

Appendix 1: characteristic features and final report of COVID-19 patients who excluded

| **Variable** | **Patient 1** | **Patient 2** | **Patient 3** | **Patient 4** | **Patient 5** | **Patient 6** | **Patient 7** | **Patient 8** |
| --- | --- | --- | --- | --- | --- | --- | --- | --- |
| **Age (year)** | 85 | 87 | 54 | 22 | 45 | 30 | 40 | 62 |
| **Gender** | Female | Female | Male | Male | Male | Male | Female | Male |
| **Body mass index (kg/m^2^)** | 23 | 29 | 26.3 | 25.7 | 30.1 | 23.5 | 32 | 29.4 |
| **Comorbidity** | Cardiovascular disease | Diabetes mellitus + Cardiovascular disease + Dementia | Diabetes mellitus + Cardiovascular disease | None | None | None | None | Diabetes mellitus + Cardiovascular disease |
| **Smoker** | No | No | Yes | Yes | No | No | Yes | Yes |
| **Drug abuser** | No | Yes | Yes | No | No | No | No | Yes |
| **Alcoholic** | No | No | No | No | No | No | No | No |
| **ASA grade** | 5 | 5 | 3 | 2 | 2 | 2 | 2 | 3 |
| **Pulmonary involvement** | Severe | Severe | Moderate to Severe | Moderate to Severe | Moderate | Moderate | Moderate | Moderate |
| **Diagnosis** | Hip fracture | Upper extremity | Lower extremity | Hip fracture | Lower extremity | Hip fracture | Hip fracture | Hip fracture + Lower extremity |
| **COVID-19 clinical symptom** | Severe dyspnea, Cough | Severe dyspnea, Cough, fever, GI symptoms | Cough, fever | Cough, fever | Dyspnea, Cough | Fever | Cough | Fever + Fatigue |
| **Laboratory findings** | Elevated CRP | Elevated ESR & CRP | Elevated ESR & CRP | Elevated ESR & CRP | Elevated CRP | Elevated CRP | Elevated CRP | Elevated CRP |
| **Radiologic findings** | Positive CT and CXR | Positive CT and CXR | Positive CT | Positive CT | Positive CT | Positive CT | Positive CT and CXR | Positive CT and CXR |
| **PCR** | Negative | Positive | Positive | Positive | Not performed | Negative | Negative | Negative |
| **Final management** | Deceased | Deceased | Delay surgery | conservative | conservative | conservative | conservative | conservative |

ASA: American Society of Anesthesiologists; PCR: Polymerase Chain Reaction; ESR: Erythrocyte Sedimentation Rate; CRP: C-Reactive Protein; CXR: chest X-ray; CT: Computed Tomography; GI: Gastrointestinal
